# Palliative Care Options for a Young Adult Patient with a Diffuse Intrinsic Pontine Glioma

**DOI:** 10.7759/cureus.1580

**Published:** 2017-08-18

**Authors:** Julian Sison, Hung Tran, Ashley Margol, Nishant Tiwari, Karla M Garcia, Jennifer Cotter, Erin Kiehna, Arthur J Olch, Kenneth Wong

**Affiliations:** 1 Robert Wood Johnson Medical School; 2 Children's Center for Cancer and Blood Diseases, Children's Hospital of Los Angeles; 3 Children's Center for Cancer and Blood Diseases, Children’s Hospital of Los Angeles; 4 Pathology and Laboratory Medicine, Children's Hospital of Los Angeles; 5 Clinical Social Work, Children's Hospital of Los Angeles; 6 Pathology and Laboratory Medicine, Children's Hospital of Los Angeles, Keck School of Medicine of the University of Southern California, Los Angeles, Ca; 7 Department of Neurosurgery, Children's Hospital of Los Angeles, Keck School of Medicine of the University of Southern California, Los Angeles, Ca; 8 Department of Radiation Oncology, Keck School of Medicine of the University of Southern California, Los Angeles, CA

**Keywords:** dipg, adolescent, young adult, palliative care, end-of-life, support, aya, psychosocial

## Abstract

Diffuse intrinsic pontine gliomas (DIPGs) are rare but devastating brain tumors that occur primarily in children. These gliomas have poor prognoses and present options focus on palliation of symptoms and prolongation of life. Here, we present a case of a 16-year-old female diagnosed with a DIPG whose age group has been mostly left out of discussions regarding psychosocial support options. This report is meant to start a conversation about the different support options available at our institution that have shown promising results in the literature for palliative care applications. These options can include camps for patients with brain tumors, psychological counseling, the Ronald McDonald House, and other psychosocial programs. Many of these programs can be tailored to meet the specific needs of adolescent and young adult (AYA) patients and will hopefully be integrated into a comprehensive palliative care regimen in future studies.

## Introduction

Brainstem gliomas are low-frequency, high-impact events that can dramatically alter the trajectory of an adolescent’s life. Diffuse intrinsic pontine gliomas (DIPGs) occur primarily in pediatric patients, with a median patient age of five to eleven years, without a gender predominance, with an incidence of 300 pediatric cases and 100 adult cases per year. These tumors can be high-grade and have a universally dismal prognosis, with a median overall survival of eight to eleven months [1]. The rarity of these tumors means that conducting randomized clinical trials is difficult and long-term survivors are rare.

The administration of palliative care can be complicated by psychosocial factors. Although there are existing recommendations when it comes to having end-of-life discussions with patients, these may not be universally effective when faced with changing contexts, such as age [2-3]. Palliative care for pediatric patients is faced with many challenges, which can include cost, availability, and timing of referrals [3]. These issues can be present for adolescent and young adult (AYA) palliative care patients, along with a host of other unique ones.

Palliative care in AYA patients is an understudied topic, so there is still a great need to explore the unique preferences of this population [4]. AYA patients present challenges as care providers must respect their emerging autonomy [5], and the need for peer support may increase as individuals progress through adolescence. AYA patients are often also treated in community hospitals that lack a palliative care expert to optimally serve their needs [3]. Few studies address these criteria in the context of palliative care, which creates a need for a discussion about how to accommodate patients’ age-related concerns. We present a case of an AYA patient with a DIPG in order to increase the awareness of the psychosocial intricacies of palliative care and to stimulate a conversation about the support options available.

## Case presentation

**Figure 1:**
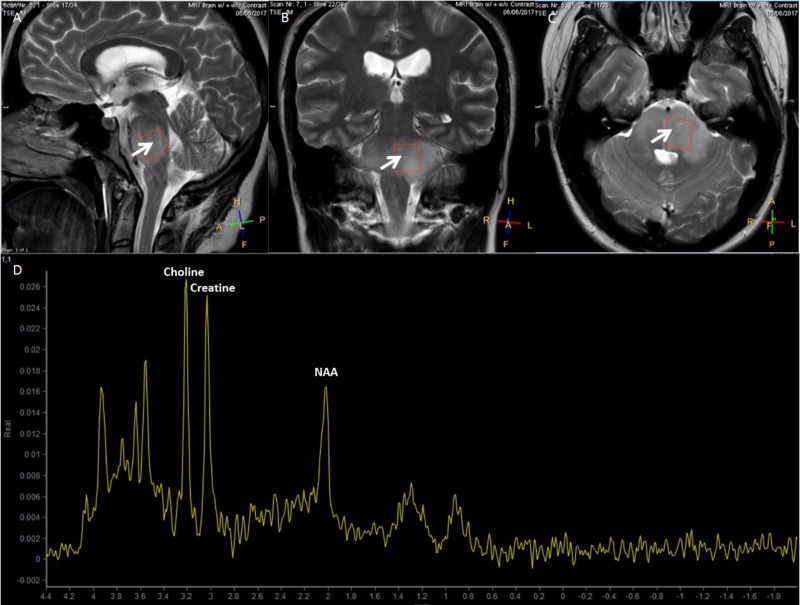
High-grade diffuse intrinsic pontine glioma in a 16-year-old female Sagittal (A), coronal (B), and axial (C) slices in T2-weighted magnetic resonance imaging showing a diffuse intrinsic brainstem hyperintense lesion (white arrows). (D) Magnetic resonance spectroscopy showing elevated choline and creatine peaks with a decreased n-acetylaspartate (NAA) peak.

**Figure 2:**
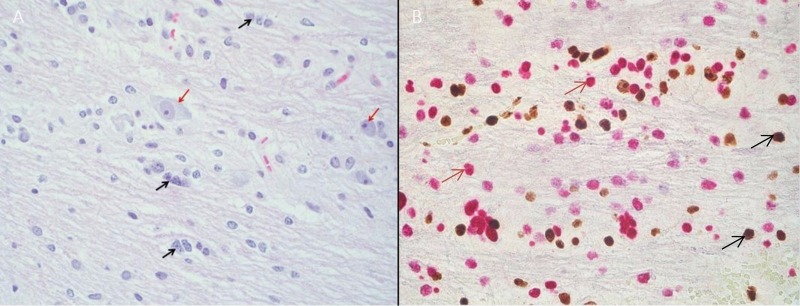
Biopsy of a diffuse intrinsic pontine glioma (A) Hematoxylin and eosin (H&E) stain showing neoplastic cells with nuclear atypia (black arrows) and intrinsic pontine neurons entrapped by neoplasm (red arrows). (B) H3K27me3 immunohistochemistry stain with wild type (black arrows) and mutant (red arrows) protein.

**Figure 3:**
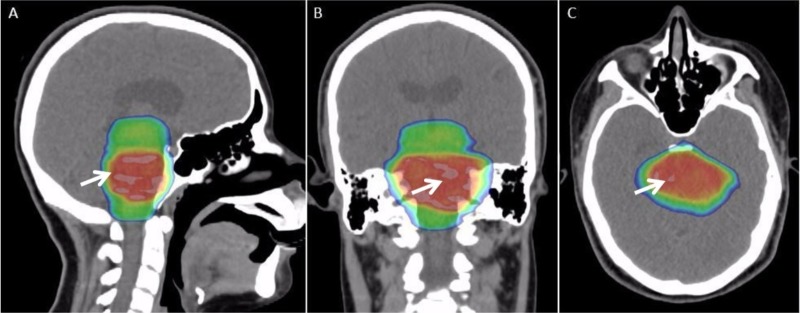
Treatment plan for a 16-year-old female with a diffuse intrinsic pontine glioma Sagittal (A), coronal (B), and axial (C) slices of a dose color wash of a 10-beam non-coplanar intensity-modulated radiation therapy (IMRT) plan (white arrows) to 54 Gy in 30 fractions.

A 16-year-old woman with a history of anxiety presented with three to four weeks of left-sided facial drooping, left-sided tongue numbness, vertigo, and difficulty closing her left eye. She had been diagnosed with Bell’s palsy and prescribed methylprednisolone for her symptoms, which did not produce relief. Magnetic resonance imaging (MRI) of the brain depicted a brainstem mass. Magnetic resonance spectroscopy (MRS) revealed elevated choline and creatine peaks with a decreased n-acetylaspartate (NAA) peak consistent with a DIPG (Figure 1A-1D). There was mild effacement of the left aspect of the fourth ventricle without hydrocephalus. Relevant family history includes thyroid cancer in her mother and aunt, and melanoma in her grandfather. Her mass was biopsied and the specimen was sent for next-generation sequencing to identify biological targets (Figure 2A, 2B). The patient was treated with a 10-beam non-coplanar intensity-modulated radiation therapy (IMRT) plan of 54 Gy in 30 fractions with an integrated boost to 60 Gy (Figure 3A-3C).

The patient’s parents were considerably upset upon hearing the diagnosis and delayed meeting to discuss psychosocial support options, creating difficulty for providers attempting to address the needs of the AYA patient. The parents did not initially want their daughter to know the full details of her diagnosis. As such, the patient was very anxious and reluctant to undergo treatment for her tumor. She required propofol sedation before gradually developing more trust in her treatment team. When her biopsy results became available, her parents agreed to meet with her physicians, therapists, nurses, and other staff to determine the best approach to provide information and support options to the patient. The patient decided to meet staff members without her parents, because she did not want them to witness her pain and distress upon receiving the news of her diagnosis.

## Discussion

Despite advances in imaging technology and radiation therapy, advances in treatment have been slower, and treatments are rarely curative, leading to the extremely poor prognosis for patients with DIPGs [1]. The reality facing most DIPG patients justifies starting a discussion about what resources are available for psychosocial support. This is particularly true for AYA patients, whose preferences so far have been mostly unaddressed.

Previous studies have surveyed different age groups regarding what principles are most valued with the hope of establishing procedures for end-of-life discussions. These surveys offer insights that can help shape the way information is delivered to patients so that it is sensitive to their needs. AYA patients in one study expressed a desire to be involved in end-of-life care discussions, but first and foremost valued being treated with respect by their care provider [6]. The results of this survey may be used to shape future guidelines regarding psychosocial support for AYA patients.

Palliative care patients may require many services beyond the bedside, including financial and emotional support. These psychosocial interventions have been shown to help patients and parents adjust to psychological stresses caused by a distressing diagnosis [7]. Since these needs can be influenced by age, it is ideal to offer services that are designed accordingly. The limited literature on this specific topic makes it worthwhile to begin a conversation about the support options that currently exist for this demographic. We will discuss here some of the different ancillary services available at our institution that may prove valuable for AYA patients.

Camps for children with cancer and their siblings have been shown to improve self-esteem and reduce anxiety [8]. The Pediatric Brain Tumor Network hosts a We Can camp with weekend retreats for brain tumor patients and their families. There are retreats specifically for AYA patients, with the option of Spanish-only camps to accommodate the large Hispanic population in Southern California. Patients participate in group therapy, socialize with peers in similar circumstances, and receive various forms of support that can cater to their requests, such as music therapy.

Adolescent support groups have been shown to increase cohesiveness between peers and with their therapists [9]. These types of programs could accommodate Spanish-speaking patients with interpreters and could pair patients by age groups. Adolescent support groups could be run on a regular basis to offer group therapy sessions to cancer patients either in person or via webcast. A psychologist and social worker can attend each session to help patients work through their issues while also allowing them to have face-to-face contact with their peers.

Burial assistance is a service offered to grieving families that may be difficult to discuss due to its morbid nature. The costs associated with funeral services are often substantial and many families may face these costs without financial assistance [10]. Families can be offered low-cost services by local funeral homes. Funeral assistance is particularly important for immigrant families interested in having services in their home country, via the more affordable cremation option. This acts as a form of financial support that may be more sensitive to the needs of patients that have travelled from other countries to receive healthcare.

The interventions listed above are suggestions for a more comprehensive set of guidelines to be designed in the future. There are many other existing psychosocial interventions that could be integrated as well, which we have compiled in Table 1. These options have delivered promising results and will hopefully pave the way for a conversation regarding the standards of palliative care in AYA patients.

**Table 1:** Various existing psychosocial support options for AYA palliative care patients The mean cost of funeral services was taken from a study investigating the cost of child death in the United States [10].

Resource	Description	Potential psychosocial benefits	Availability
We Can Camp	Weekend retreats for brain tumor patients and their families.	Camps are offered in Spanish and there are specific retreats for teen/young adult patients. Grief camps can help parents cope with the loss of a child.	Local
School Transition and Re-entry (STAR) Service	Helps parents of pediatric cancer patients navigate the school system and advocate for their child’s needs to prevent patients from falling behind in school.	Can keep patients in the same grade at school, allowing them to continue attending classes with students of the same age. Can advocate for patients who may require Special Education, allowing them to receive an education appropriate to their abilities and needs following treatment.	Local
Psychological counseling	Staff psychologists at the hospital can offer counseling to patients based on their emotional and psychosocial needs.	Expert psychologists can address the concerns unique to different ages.	National
Ronald McDonald House	Affordable housing for both patients and their families in proximity to the hospital. Makes long-term stays for patients more affordable and can limit the commute for families that have to attend daily radiation treatments.	Enables patients to continue to live near their families/support system.	National
Social work	Assists patients and their families by providing a host of services, including evaluations, crisis intervention, financial planning, and patient advocacy for medical and other services.	Can deal with a range of financial, emotional, and logistical concerns that may be specific to age.	National
Music/Expressive Arts therapy	Local artists and certified Expressive Arts therapists help patients develop coping strategies and normalize the hospital environment for patients.	Therapists can accommodate many musical preferences, which may be dependent on patients’ ages.	National
Let's Hang Out/Teen Scene	A group to connect and help patients relate to others. Topics discussed are rotated, and there are age-specific sessions.	Caters to concerns of teens/young adults via age-specific sessions.	Local
Look Good Feel Better	A class hosted by the American Cancer Society to teach teenage patients to use makeup to deal with their changes in appearance due to treatment.	Helps teen patients deal with some of the physical changes associated with cancer treatment.	National
Hematology-Oncology Psychosocial and Education (HOPE) Resource Center	A central location with informational booklets and staff to help families navigate the many support options available. The room is always unlocked so self-service is available.	Patients and their families can be better equipped to select from the variety of support options available. Patients can select as few or as many programs as they desire.	Local
Veteran Parent Program	Provides parent-to-parent mentoring by matching parents of recently diagnosed children with trained parent volunteers.	Parents can be paired with others that share their circumstances and can more effectively understand their concerns.	Local
Funeral assistance	Families are advised on affordable burial/cremation options and are directed toward affordable local funeral homes for services.	In Southern California, funeral services normally cost upwards of $6000. Families can save thousands of dollars if directed toward local funeral homes that offer reduced fees in the event of a child’s death, or by choosing cremation. As a result of these discounts, the mean estimated cost of funeral services in the United States is $2,419.	National

## Conclusions

Despite the medical advances in imaging, diagnosis, and treatment in comprehensive cancer care, DIPGs still have a dismal prognosis. This grim diagnosis has the potential to disrupt many aspects of a patient’s life and may produce a need for support beyond the bedside. The nature of support can vary with age, as in the case of the AYA patient with a DIPG presented here. We have outlined different options that have shown promising results in previous studies, including camp retreats and adolescent support groups. These may serve as model support systems for future AYA palliative care patients and will hopefully make their situations less devastating. However, the dearth of literature about this topic indicates a need for further studies to establish more formal guidelines when caring for palliative patients of this demographic. These guidelines may incorporate the psychosocial options mentioned here to properly address the needs of AYA patients.
